# A Multidisciplinary Care Pathway for Childhood Mouth Breathing: The Pediatric Allergist, ENT, and Orthodontic Perspective

**DOI:** 10.3390/medicina62101940

**Published:** 2026-10-08

**Authors:** Luca Pecoraro, Martina Attanasi, Francesca Marra, Matteo Romanello, Franco Ferrante, Flavia Indrio

**Affiliations:** 1 Department of Experimental Medicine Pediatric Section, University of Salento Hospital “Vito Fazzi”, 73100 Lecce, Italy; flavia.indrio@unisalento.it; 2 Pediatric Department, University of Bari Aldo Moro, 70121 Bari, Italy; 3 ENT Unit, Hospital “Vito Fazzi”, 73100 Lecce, Italy; 4 Dentist, Specialist in Oral Surgery, Private Practitioner, 73100 Lecce, Italy

**Keywords:** pediatric mouth breathing, nocturnal mouth breathing, sleep-disordered breathing, primary snoring, multidisciplinary care pathway

## Abstract

*Background*: Mouth breathing (MB) in children is a functional alteration characterized by the substitution of nasal breathing with oral or mixed breathing. Persistent MB, particularly when occurring during sleep (nocturnal MB), may be associated with sleep fragmentation, neurobehavioral disturbances, and alterations in craniofacial development. Although established sleep-disordered breathing (SDB), particularly obstructive sleep apnea syndrome (OSAS), is well-recognized and addressed by international guidelines, children presenting with persistent or nocturnal MB without established diagnostic criteria for OSAS may remain outside conventional diagnostic pathways. The multifactorial nature of MB therefore requires the coordinated evaluation of nasal, allergic, upper airway, craniofacial, dental, and orofacial factors. *Methods*: This narrative review was based primarily on a structured search of the PubMed/MEDLINE database covering publications from database inception to June 2026, supplemented by manual screening of the reference lists of relevant articles and clinical guidelines. The literature was selected according to relevance to the review objectives, methodological robustness, clinical applicability, and contribution to pediatric, allergological, otorhinolaryngological, and dental/orthodontic perspectives. Priority was given to systematic reviews, meta-analyses, randomized controlled trials, clinical practice guidelines, consensus documents, and high-quality observational studies. A total of 79 references were included in the final manuscript. *Results*: The reviewed evidence indicates that MB may be associated with allergic and nasal conditions, upper airway obstruction, craniofacial and dental abnormalities, and orofacial functional alterations, and may coexist with SDB in selected children. The evidence was synthesized across pediatric, allergological, otorhinolaryngological, and orthodontic perspectives to develop a multidisciplinary care pathway based on the clinical identification of MB, targeted specialist assessment, objective evaluation of SDB when indicated, and individualized management and follow-up. *Conclusions*: MB may represent an important clinical entry point for the early recognition of potentially treatable conditions and associated SDB. The proposed multidisciplinary care pathway integrates pediatric, allergological, otorhinolaryngological, and dental/orthodontic expertise to support coordinated assessment, individualized management, and longitudinal follow-up of children with MB.

## 1. Introduction

Mouth breathing (MB) is defined as the substitution of nasal breathing with oral or mixed breathing [1]. In clinical practice, this broad definition encompasses different patterns that may vary in their frequency, persistence, and timing. Among these, persistent and nocturnal MB are particularly relevant because recurrent oral or mixed breathing may indicate an underlying nasal, upper airway, functional, or sleep-related condition. Persistent MB may therefore be used as a clinical descriptor of a recurrent breathing pattern that extends beyond transient or acute episodes and may occur during wakefulness, sleep, or both. Nocturnal MB is typically identified through parental or caregiver report of mouth-open breathing during sleep and may be supported, when available, by direct observation or home video recordings. Clinical assessment should distinguish predominantly habitual or functional MB from MB related to nasal obstruction or suspected sleep-disordered breathing (SDB) based on nasal patency, allergic or upper airway conditions, and relevant nocturnal and daytime symptoms. Persistent MB without clear nasal obstruction may suggest a functional component, whereas features of nasal obstruction or SDB should prompt targeted assessment. MB should therefore be considered a clinical finding requiring etiological evaluation rather than a synonym for SDB or OSAS [2,3,4]. It is frequently associated with SDB, a spectrum of disorders characterized by increased upper airway resistance and impaired airway patency during sleep [3,4]. Pediatric SDB ranges from primary snoring and other mild forms to obstructive sleep apnea syndrome (OSAS), with an estimated prevalence of 4–11% [3,5]. While OSAS is relatively well-characterized, milder phenotypes associated with MB remain less well-defined and are often underestimated in clinical practice. In general, MB is common in childhood, with a recent systematic review and meta-analysis reporting a pooled prevalence of 21.1% among children aged 3–18 years (95% CI, 13.1–30.4%) [6]. These estimates refer to MB irrespective of the time of occurrence and cannot be directly extrapolated to nocturnal MB. The epidemiology of MB is less consistently characterized, partly because of differences in definitions and assessment methods. In particular, the extent to which MB remains unrecognized in routine clinical practice has not been systematically quantified. MB is a multifactorial condition that may result from persistent nasal obstruction related to AR, adenotonsillar hypertrophy, turbinate enlargement, or structural abnormalities of the nasal and oral cavities [2]. On the other hand, causes related to the mouth are associated with alterations in craniofacial growth, dental occlusion, tongue posture, and orofacial muscle function, highlighting the complex interaction between airway function and craniofacial development. Despite its frequent occurrence in childhood, MB is often considered a benign habit rather than a clinical condition requiring systematic evaluation. Evidence specifically addressing the clinical consequences of isolated MB remains limited, and much of the available literature focuses on children with SDB or OSAS [1,7,8,9,10,11,12,13,14]. In contrast, a growing body of literature has described sleep fragmentation and neurocognitive and behavioral alterations in children with primary snoring and other forms of SDB, even in the absence of established OSAS [15,16,17,18,19,20,21,22,23,24,25,26,27,28]. Although these studies did not specifically investigate isolated MB, their findings may be clinically relevant to children with MB when mouth breathing occurs in association with nocturnal respiratory symptoms or other features suggestive of SDB. This is particularly relevant during preschool and school-age years, when sleep quality plays a critical role in neurodevelopment, learning, and behavior. Specifically, an association between primary snoring and reduced cognitive performance has been demonstrated across multiple domains, including intelligence, attention, executive function, language, memory, and visuospatial abilities. Notably, neurocognitive impairments have been reported across the entire SDB spectrum, including in children with primary snoring. Furthermore, the magnitude of IQ reduction in children with primary snoring has been reported to be comparable to that observed in mild OSAS [22]. Uema et al. showed that children with primary snoring performed worse on learning tests not only compared with healthy controls, but also compared with children with OSAS [23]. Norman et al. reported that snoring is associated with multiple movement-related arousals, resulting in greater sleep fragmentation than apnea alone [24]. This may explain why children with primary snoring, despite the absence of gas exchange abnormalities or oxygen desaturation typical of OSAS, may still experience significant sleep disruption and reduced REM sleep. This, in turn, may negatively affect neurocognitive processes such as long-term memory consolidation and may have relevant implications for academic performance [22,25]. The preschool and school-age periods represent critical stages of neurodevelopment characterized by synaptic maturation and the acquisition of cognitive and intellectual abilities. These processes may be compromised by poor sleep quality due to unrecognized or untreated breathing disorders [26]. Consequently, cognitive processing, learning, and knowledge consolidation, particularly in school settings, may be impaired in children with primary snoring [17]. Moreover, affected children may present reduced inhibitory control and increased impulsivity compared with their non-snoring peers, contributing to deficits in attention and executive functioning [27]. The Pediatric Sleep Questionnaire (PSQ) represents a useful clinical screening tool for identifying children at risk of SDB and may help characterize symptom burden beyond clinically overt OSAS. A study by Gileles-Hillel et al. found that a positive PSQ was associated with several comorbidities, including asthma, trisomy 21, prematurity, developmental delay, attention deficit disorder, chronic lung disease, congenital heart disease, and elevated BMI. Approximately one-third of children with mild SDB exhibited persistent symptoms up to two years after diagnosis, highlighting the importance of systematic assessment and longitudinal follow-up, particularly in children with recognized risk factors [29]. In light of these findings, the early identification of children with nocturnal MB and nocturnal symptoms is important. The multifactorial nature of MB requires an integrated clinical assessment considering not only nocturnal symptoms, but also nasal patency, allergic disease, adenotonsillar obstruction, craniofacial morphology, dental occlusion, and orofacial function. Multidisciplinary evaluation may improve the identification and management of the multiple factors contributing to pediatric SDB [2,30,31]. A coordinated approach involving pediatricians, pediatric allergists, ENT specialists, and orthodontists is therefore essential. Current guidelines primarily address clinically overt OSAS and provide limited guidance for subtle forms such as MB [3,30,31,32]. Moreover, the lack of standardized definitions and the difficulty in distinguishing primary snoring from OSAS contribute to the underdiagnosis of milder phenotypes and limit the interpretation of existing evidence [32]. The aim of this review is to analyze the complementary roles of pediatric allergists, ENT specialists, and orthodontists in the management of these patients and to propose a practical diagnostic multidisciplinary care pathway applicable in both clinical and research settings.

## 2. Materials and Methods

**Figure 1:**
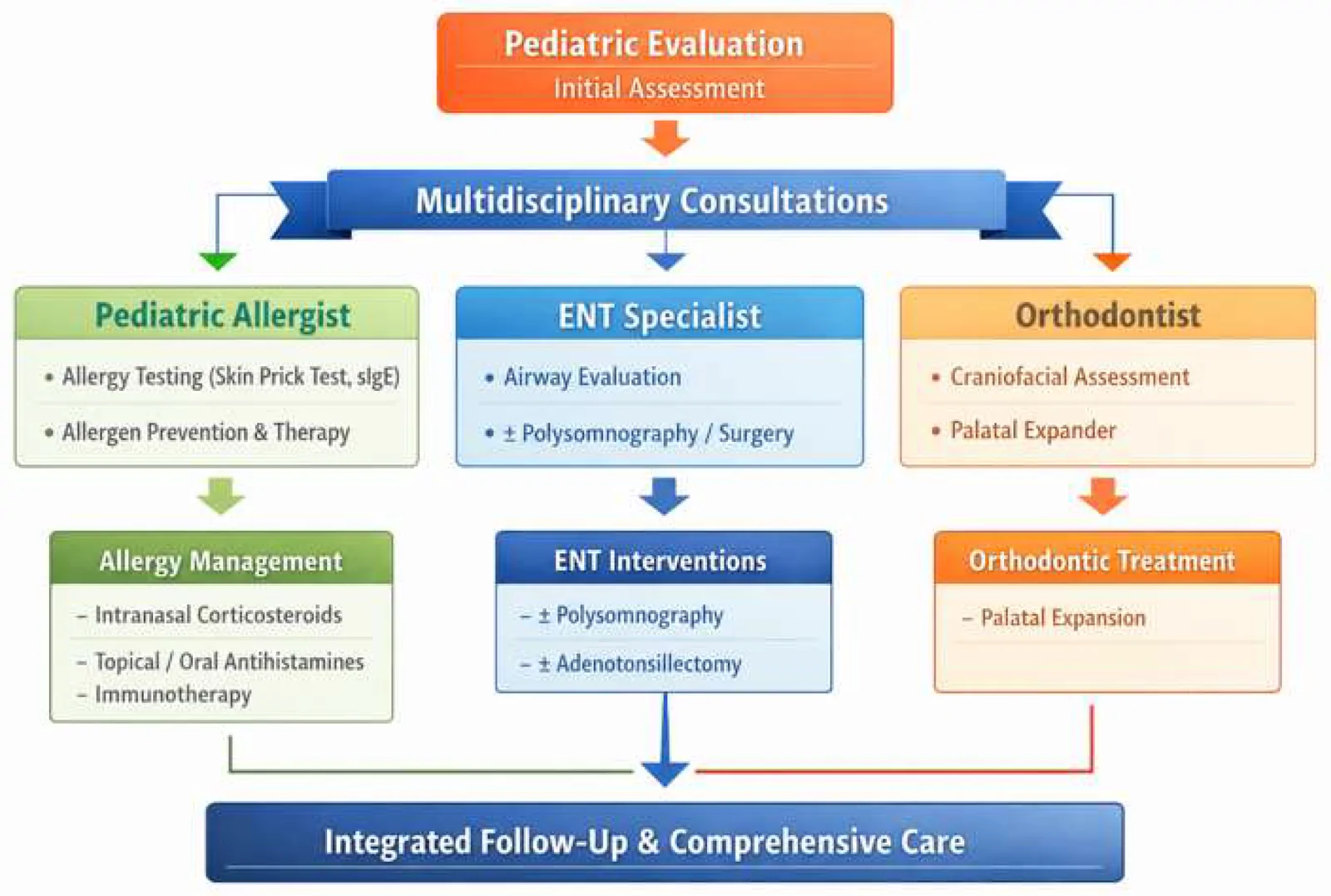
Multidisciplinary care pathway for pediatric mouth breathing.

A structured literature search was performed in the PubMed/MEDLINE database, covering publications available from database inception to June 2026. The search strategy combined free-text terms and related keywords using Boolean operators (AND/OR). Because this was a narrative review addressing several complementary clinical domains, no single fixed search string was applied to all topics; instead, predefined combinations of terms were used according to the specific clinical question. Representative combinations included (“mouth breathing” OR “nocturnal mouth breathing”) AND (“children” OR “pediatrics”); (“mouth breathing” OR “nocturnal mouth breathing”) AND (“sleep-disordered breathing” OR “primary snoring” OR “obstructive sleep apnea”); (“mouth breathing”) AND (“allergic rhinitis” OR “adenoid hypertrophy” OR “tonsillar hypertrophy” OR “turbinate hypertrophy”); and (“mouth breathing” OR “sleep-disordered breathing”) AND (“orofacial myofunctional therapy” OR “rapid maxillary expansion” OR “orthodontics”). Additional combinations were used when appropriate to address specific aspects of pediatric MB and SDB. Reference lists of relevant articles and guidelines were also manually screened to identify additional studies not retrieved through the primary database search. No restrictions were applied regarding publication language or publication date. Studies involving pediatric populations with MB, nocturnal respiratory symptoms, SDB, primary snoring, upper airway obstruction, allergic or nasal disease, or craniofacial, dental, and/or orofacial abnormalities that are potentially associated with altered breathing patterns were considered relevant. Studies addressing exclusively adult populations or unrelated populations, conditions, or outcomes were excluded. Priority was given to systematic reviews, meta-analyses, randomized controlled trials, clinical practice guidelines, consensus documents, and high-quality observational studies. Study selection was based on predefined criteria of relevance to the review objectives, methodological robustness, clinical applicability, and contribution to the pediatric, allergological, otorhinolaryngological, or orthodontic perspectives. Methodological robustness was assessed qualitatively according to the appropriateness of the study design and methods, clarity of the study population and outcomes, and consistency of the reported findings. Clinical applicability was assessed according to the relevance of the population, diagnostic or therapeutic approach, and outcomes to pediatric MB and SDB and to the clinical questions addressed by the proposed multidisciplinary pathway. Particular emphasis was placed on studies addressing the clinical consequences of MB and SDB, diagnostic strategies, nasal and upper airway obstruction, allergic disease, craniofacial and dental abnormalities, multidisciplinary management, and follow-up. The retrieved literature was evaluated by the authors according to these criteria. When the relevance or interpretation of individual studies was uncertain, eligibility was discussed among the authors until agreement was reached. The multidisciplinary care pathway proposed in this review was developed through a structured narrative synthesis of the retrieved literature, integrating evidence from pediatric, allergological, otorhinolaryngological, dental, and orthodontic sources with recommendations from currently available clinical guidelines and consensus documents. Accordingly, the proposed pathway integrates the complementary expertise of pediatric allergists, ENT specialists, and dentists or orthodontists to provide a practical framework for initial assessment and appropriate specialist referral, rather than representing a new multidisciplinary care pathway for OSAS. The pathway therefore represents an evidence-informed framework developed by the authors rather than the result of a formal Delphi or other structured consensus methodology. As this was a narrative review, no formal systematic-review screening process or PRISMA flow diagram was undertaken. The number of records initially retrieved was therefore not prospectively recorded as part of a quantitative screening workflow. The final reference selection was based on the relevance and quality criteria described above, with particular emphasis on studies directly informing the clinical assessment, differential diagnosis, multidisciplinary management, and follow-up of children with MB and associated SDB. Following the revision of the manuscript, 79 references were included in the final version. Figure 1 was generated using OpenAI ChatGPT (GPT-5.6 Luna) image-generation tools and subsequently reviewed, edited, and validated by the authors for scientific accuracy.

## 3. The Pediatric Allergist Perspective

### 3.1. Allergic Inflammation and the Relationship with MB and SDB

Allergic rhinitis (AR) is an IgE-mediated inflammatory condition triggered by specific inhaled allergens such as dust mites, pollens, molds, and animal dander. It has a prevalence of 19.87% and is characterized by early- and late-phase inflammatory responses [33,34,35,36]. These responses lead to nasal mucosal inflammation and edema, resulting in the characteristic symptoms of AR, including rhinorrhea, nasal congestion, sneezing, and pruritus [34,37,38,39,40,41]. In clinical practice, the coexistence of AR and sleep-disordered breathing (SDB) is frequent. A meta-analysis by Cao et al. demonstrated that children with SDB have a higher prevalence of AR compared with those without SDB, reporting a prevalence of 41–45% among children with SDB/OSA and odds of AR 2.12 times higher than in controls [42]. However, most studies addressing the relationship between AR and sleep disorders have focused on more severe phenotypes, particularly OSAS, leaving limited evidence regarding milder manifestations such as MB [43,44]. The relationship among AR, snoring, and SDB has been widely investigated, with AR identified as a major risk factor for pediatric snoring and an associated contributor to obstructive sleep apnea [43,45,46,47,48]. Nasal inflammation and airway narrowing may increase upper airway resistance, thereby promoting snoring [46]. AR is associated with increased nasal airflow resistance and represents an important contributor to nocturnal snoring and oral breathing patterns in children [45,49]. Although these findings provide a rationale for considering AR in children with MB, they should not be directly extrapolated to children with isolated MB or mild SDB, as the available evidence in these populations remains limited [42,43,44]. In such children, AR should therefore be considered a potentially modifiable contributor to nasal obstruction and MB rather than an established causal factor, supporting individualized allergological assessment when clinical features suggest an allergic component. Although symptoms may appear mild, their clinical impact should not be underestimated, as nasal obstruction can significantly impair sleep quality, cognitive function, and overall performance, particularly in school-aged children. This may result in fatigue, reduced attention span, and difficulties in learning and memory [50,51,52,53,54]. A cross-sectional study by Tung et al. reported shorter telomere length in patients with a history of snoring and atopic diseases, particularly asthma and AR. These findings suggest that snoring in young atopic patients may reflect poor disease control and may be associated with accelerated telomere shortening, potentially driven by chronic oxidative stress and consequent DNA damage [55].

### 3.2. Allergological Assessment

Appropriate assessment of AR is important in children with persistent or nocturnal MB, particularly when clinical features suggest an allergic component. Diagnosis is based on the correlation between clinical history and diagnostic testing. Symptoms include rhinorrhea, pruritus, sneezing, and nasal obstruction, often accompanied by lacrimation, cough secondary to post-nasal drip, and sleep disturbances. Diagnosis relies on demonstrating allergen-specific IgE through skin prick testing (SPT) and serum IgE assays, interpreted in conjunction with the clinical history and phenotype [56]. Skin prick testing can also be performed in children under five years of age; positivity at age five may predict later sensitization and is associated with respiratory conditions such as allergic rhinoconjunctivitis and recurrent wheezing in later childhood and adolescence, although a negative result does not exclude future sensitization or disease development [57]. Additional diagnostic tools include serum-specific IgE assays and nasal provocation tests [50,58]. AR is frequently associated with other atopic conditions, including allergic conjunctivitis, atopic dermatitis, and asthma, which should be actively investigated. Moreover, inflammation of the nasal mucosa may extend to adjacent structures, contributing to nasal polyps, otitis media, adenoidal hypertrophy, chronic sinusitis, malocclusion, dental and skeletal misalignment, and adenoid facies [56].

### 3.3. Therapeutic Management

According to the 2024 Delphi Consensus of the Italian Society of Pediatric Allergy and Immunology (SIAIP), topical corticosteroids are effective and safe for treating AR, as they rapidly reduce inflammation and improve nasal obstruction and associated comorbidities. There was also near-complete agreement that the combination of antihistamines and corticosteroids is highly effective and well-tolerated in pediatric patients, with rapid onset of action [59]. According to ARIA guidelines, intranasal corticosteroids represent first-line therapy for patients with persistent or moderate-to-severe symptoms. Second-generation oral or intranasal antihistamines are recommended for symptoms such as sneezing and itching. Combination therapy may be used in patients with inadequate response to monotherapy, while allergen immunotherapy may be considered in refractory cases [60].

## 4. The ENT Perspective

### 4.1. Upper Airway Obstruction and Pathophysiology

The otorhinolaryngologist plays a central role in evaluating pediatric MB, particularly when upper airway obstruction is suspected. The upper airway is a dynamic anatomical system in which nasal airflow resistance, lymphoid tissue, craniofacial morphology, and neuromuscular control interact to maintain airway patency. Persistent nasal obstruction may increase upper airway resistance and promote oral or mixed breathing, while chronic alterations in breathing patterns may contribute to changes in tongue posture, lip competence, and craniofacial development. Adenotonsillar hypertrophy represents one of the most common causes of upper airway obstruction in children. During sleep, the reduction in upper airway muscle tone may further increase airway collapsibility, particularly in children with anatomical narrowing. Other contributing factors include turbinate hypertrophy, nasal septal abnormalities, craniofacial restriction, obesity, neuromuscular disorders, and syndromic conditions. Craniofacial abnormalities are strongly associated with an increased risk of SDB. Syndromic conditions such as Pierre Robin sequence show SDB prevalence ranging from 10% to 87.5% compared with approximately 2–4% in the general population. Micrognathia is strongly associated with an increased risk of OSA in children, while Down syndrome presents multiple risk factors, including macroglossia, generalized hypotonia, and midface hypoplasia [61].

### 4.2. Clinical and Objective Assessment

Comprehensive history-taking should assess nocturnal symptoms, including habitual snoring (>3 nights per week), witnessed apneas, nocturnal MB, restless sleep, and secondary enuresis, as well as daytime manifestations. Unlike adults, pediatric OSA is more commonly associated with behavioral disturbances such as hyperactivity and impulsivity rather than excessive daytime sleepiness [62]. Physical examination should include tonsillar assessment using the Brodsky classification (grades 0–4), evaluation of adenoidal facies, nasal patency, and Mallampati scoring. Nasal endoscopy is the preferred diagnostic modality for adenoid evaluation, allowing for direct visualization without radiation exposure and providing greater diagnostic accuracy than lateral neck radiography. The Cassano endoscopic classification grades adenoid hypertrophy according to the percentage of choanal obstruction [63]. Overnight polysomnography (PSG) remains the reference standard for the objective diagnosis and severity assessment of pediatric OSA. According to ICSD-3 criteria, diagnosis requires compatible clinical symptoms together with either at least one obstructive respiratory event per hour of sleep or evidence of obstructive hypoventilation. The obstructive apnea–hypopnea index (OAHI) is commonly used to characterize disease severity, with mild disease generally defined as an OAHI of 1–5 events/hour, moderate disease as 5–10 events/hour, and severe disease as >10 events/hour. Preoperative PSG is particularly relevant in children at increased risk of perioperative complications or when the clinical indication for surgery is uncertain [30]. In children presenting primarily with persistent MB, particularly nocturnal MB, PSG should therefore be considered based on the clinical suspicion of associated SDB rather than as a mandatory first-line investigation for MB itself. The recently published Canadian Thoracic Society/Canadian Sleep Society guideline confirms polysomnography as the diagnostic standard for pediatric OSA, while supporting the Pediatric Sleep Questionnaire (PSQ) and overnight oximetry as adjunctive tools rather than diagnostic tests [64]. MB and OSA are not synonymous, and the presence of nocturnal MB alone does not establish a diagnosis of OSA. The ENT assessment should initially identify potential anatomical causes of persistent oral breathing and determine whether clinical history suggests clinically relevant sleep-related obstruction. Objective sleep assessment can then be used to characterize the severity of SDB when indicated. Drug-induced sleep endoscopy (DISE) provides a dynamic, three-dimensional assessment of the sites and patterns of upper airway collapse during pharmacologically induced sleep. It is particularly useful in children with persistent OSA after adenotonsillectomy, in whom identification of the residual site of obstruction is essential for selecting further treatment. An international survey reported broad support for the use of DISE in children with persistent OSA after adenotonsillectomy [65]. In children with persistent MB, DISE should therefore be reserved for selected cases in which persistent sleep-related obstructive symptoms warrant further assessment rather than being considered a routine investigation of MB.

### 4.3. Therapeutic Management

Management should be individualized according to the underlying cause of MB, the presence and severity of associated SDB, comorbidities, and the clinical consequences of persistent oral breathing. When MB is primarily related to nasal obstruction, treatment should be directed toward restoring nasal patency. When adenotonsillar hypertrophy represents the main anatomical cause of clinically significant SDB, surgical treatment may be indicated. Conversely, when obstruction is limited or symptoms are mild, conservative management and clinical reassessment may be appropriate. Evidence from the Childhood Adenotonsillectomy Trial (CHAT) demonstrated spontaneous normalization of polysomnographic findings in approximately 46% of children assigned to watchful waiting at follow-up, although adenotonsillectomy produced greater improvements in behavioral, quality-of-life, and polysomnographic outcomes [66,67]. More recent evidence from the Pediatric Adenotonsillectomy Trial for Snoring (PATS) also supports the possibility of clinical improvement with conservative management in selected children with mild SDB [68]. In children presenting with nocturnal MB, the decision to observe or intervene should therefore be based on the underlying cause, symptom burden, evidence of SDB, and associated morbidity, with clinical reassessment when symptoms persist. When clinically significant OSA is documented in association with adenotonsillar obstruction, adenotonsillectomy remains the main surgical treatment. Tonsillectomy and adenoidectomy can substantially improve symptoms and polysomnographic parameters, although residual or recurrent SDB may occur, particularly in children with obesity, Down syndrome, craniofacial abnormalities, severe baseline disease, or other comorbidities. Tonsillotomy has demonstrated efficacy comparable to total tonsillectomy in selected children, with the additional advantages of reduced postoperative hemorrhage, faster recovery, and lower analgesic requirements, although a slightly higher recurrence rate has been reported [69]. Adenoidectomy alone may be appropriate in selected non-obese children younger than 7 years with an OAHI < 10 events/hour and small tonsils (Brodsky < 3), with outcomes comparable to adenotonsillectomy in this subgroup [3,70,71,72,73]. A major clinical challenge is persistent MB after resolution or improvement of adenotonsillar obstruction. In children with reduced maxillary transverse dimensions, rapid palatal expansion may be indicated [73]. In other cases, persistence may reflect neuromuscular adaptation, with long-standing oral posture patterns involving the orofacial musculature. In this context, orofacial myofunctional therapy (OMT) may play a role. OMT consists of targeted isotonic and isometric exercises involving the tongue, lips, and facial muscles [74]. From an ENT perspective, its aim includes restoring tongue posture against the hard palate, improving lip seal by strengthening the orbicularis oris muscle, and re-educating nasal breathing patterns. Clinical evidence suggests that OMT may reduce residual AHI after surgery. It may be considered in selected children with persistent functional abnormalities, although evidence regarding its efficacy for pediatric SDB remains limited and heterogeneous [75]. By improving the function of upper airway dilator muscles, OMT may act as a functional stabilizer and reduce airway collapse during sleep. Importantly, treatment of MB should not be equated with treatment of OSA. When MB persists after the correction of nasal or adenotonsillar obstruction, the clinical objective is to identify the factors that maintain the oral breathing pattern. These may include persistent nasal obstruction, residual upper airway obstruction, altered tongue posture, impaired lip seal, craniofacial abnormalities, dental malocclusion, or orofacial dysfunction. Accordingly, ENT reassessment should be integrated with allergological and dental/orthodontic evaluation, with OMT considered when functional abnormalities are identified. Overall, the ENT evaluation of a child with persistent MB, particularly nocturnal MB, should proceed from identification of the underlying anatomical and functional causes to assessment of possible associated SDB, and when indicated, objective characterization with PSG. Existing guidelines provide an essential framework for the diagnosis and treatment of clinically significant SDB and OSA, but they do not specifically address MB as an early or isolated clinical presentation. It is fundamental to avoid both the under-recognition of clinically relevant SDB and unnecessary investigation or overtreatment of children whose MB is not associated with clinically significant OSA.

## 5. The Orthodontic Perspective

### 5.1. Clinical Recognition of MB in the Context of Craniofacial Assessment

Orthodontists play a crucial role as frontline professionals in the early identification of SDB in both pediatric and adult populations [76,77,78]. Clinical recognition of MB may be improved by combining visual assessment, targeted questions regarding nocturnal symptoms and nasal obstruction, and simple functional breathing assessments [6]. Pediatric dental evaluations often occur during the developmental window in which SDB and MB are most prevalent, making early recognition particularly relevant. Accordingly, dental professionals should be trained to identify early clinical signs of MB and understand their potential systemic and functional implications [76,77,78]. Several intraoral and craniofacial features should raise clinical suspicion, including persistent MB, predominantly nocturnal MB, skeletal Class II relationship, high-arched or constricted palate, tonsillar or adenoidal hypertrophy, macroglossia, convex facial profile, nocturnal bruxism, and dental crowding. When these red flags are identified, the orthodontist plays a central role in the early recognition of children who may require further evaluation for SDB or for craniofacial and dental factors that may contribute to MB [76,77,78]. This process includes not only clinical examination but also a targeted medical history, with parental questioning regarding habitual snoring, nocturnal MB, witnessed apneas, restless sleep, daytime behavioral disturbances, and academic difficulties.

### 5.2. Multidisciplinary Assessment and Functional Determinants

When clinical suspicion is present, timely referral within a multidisciplinary network, including pediatricians, pediatric allergists, pulmonologists, otorhinolaryngologists, and speech-language therapists, is essential to distinguish isolated or predominantly functional MB from MB associated with nasal or upper airway obstruction and clinically relevant SDB. Specifically, a multidisciplinary study of children with persistent MB further demonstrated the frequent coexistence of allergic, adenotonsillar, orofacial, dental, and speech-related abnormalities, supporting the value of coordinated assessment across specialties [79]. This distinction is particularly important because treatment of the craniofacial component should be directed toward children in whom a specific anatomical or functional abnormality is identified. In selected patients, orthodontists may also contribute directly to the management of persistent MB through functional orthodontic approaches aimed at correcting craniofacial abnormalities that may contribute to upper airway narrowing.

### 5.3. Orthodontic Management

Rapid maxillary expansion (RME) is particularly relevant when MB coexists with a documented transverse maxillary deficiency, such as a narrow maxillary arch or posterior crossbite. The presence of MB or nocturnal MB alone, in the absence of a documented transverse maxillary deficiency, should not be considered an indication for RME. The potential benefit of maxillary expansion is greatest when a true transverse skeletal discrepancy is present, and treatment is undertaken during active craniofacial growth. RME is therefore most commonly considered during the mixed dentition or early permanent dentition, when the midpalatal suture remains sufficiently responsive to orthopedic expansion. In more skeletally mature adolescents, increasing resistance of the midpalatal suture may reduce the orthopedic effect of conventional RME, and alternative expansion approaches may need to be considered according to individual skeletal maturity and orthodontic assessment. Thus, patient age should be interpreted together with dental and skeletal maturation rather than used as an isolated criterion for treatment selection. When appropriately indicated, RME increases maxillary transverse dimensions and may improve nasal airflow and upper airway dimensions while also facilitating a more physiological tongue position within the expanded palatal space. These effects may be particularly relevant in children with MB associated with maxillary constriction. The potential respiratory benefit of RME should nevertheless be interpreted as correcting an underlying transverse craniofacial abnormality rather than as a direct treatment for MB or OSAS. Orthodontic intervention is therefore not limited to dental alignment but may contribute to functional optimization of the upper airway in appropriately selected children. In pediatric patients with Class II skeletal patterns associated with mandibular retrognathia, functional appliances may be indicated during active growth. Common devices include monobloc, bionator, twin-block, Frankel, Andresen, Herbst, Bimler, Harvold, Herren, and Woodside appliances, all aimed at mandibular advancement and potentially improving upper airway dimensions. Their use should be individualized according to the underlying skeletal pattern, the stage of craniofacial development, dental and skeletal relationships, and the presence of clinically relevant respiratory symptoms. In children with MB, these interventions should therefore be considered only when mandibular retrognathia represents a relevant craniofacial component of the breathing disorder. One of the distinctive features of pediatric SDB is the potential for substantial improvement or resolution with early diagnosis and timely intervention. Unlike adults, in whom obstructive sleep apnea is often chronic and requires long-term management, children may experience improvement following targeted orthodontic, functional, or surgical treatment. However, orthodontic treatment should be directed by the specific anatomical or functional abnormality identified during the multidisciplinary assessment rather than by the presence of MB alone. From an orthodontic perspective, the primary objective is therefore to identify craniofacial and dental abnormalities that may contribute to MB and to determine whether a specific intervention is indicated. Early orthodontic assessment may facilitate the identification of children with transverse maxillary deficiency who are candidates for maxillary expansion during favorable stages of craniofacial development, while functional orthopedic appliances may be considered during active growth in children with clinically relevant mandibular retrognathia. These interventions should be integrated with the management of nasal and upper airway obstruction, and when indicated, orofacial myofunctional therapy, rather than being considered isolated treatments for MB or OSAS [76,77,78].

## 6. The Multidisciplinary Perspective

MB in children is often underestimated, and persistent MB, particularly when occurring during sleep, may have relevant clinical implications. Rather than being considered a benign habit, persistent MB, particularly when occurring during sleep, may be associated with sleep fragmentation, neurocognitive impairment, behavioral disturbances, and alterations in craniofacial development. Its etiology is multifactorial, including allergic inflammation, structural upper airway obstruction, and dental or craniofacial abnormalities, underscoring the need for a multidisciplinary approach to support comprehensive assessment and individualized management. Several international guidelines provide diagnostic pathways for pediatric sleep-disordered breathing (SDB) and obstructive sleep apnea syndrome (OSAS). For example, the American Academy of Pediatrics recommends systematic screening for snoring, and in children with habitual snoring and clinical features suggestive of OSAS, objective evaluation with polysomnography or referral to a specialist [30]. However, these recommendations primarily address the recognition, diagnosis, and management of established or suspected SDB/OSAS rather than providing a dedicated pathway for children presenting with persistent MB, particularly nocturnal MB, without established diagnostic criteria for OSAS. The individual diagnostic and therapeutic components of the proposed pathway, including the assessment of allergic disease and nasal obstruction, ENT evaluation of adenotonsillar obstruction, polysomnography when clinically indicated, and assessment of craniofacial and dental abnormalities, are based on previously published evidence and current clinical recommendations. The pathway (Figure 1) begins with a comprehensive pediatric assessment to identify MB, nocturnal symptoms, and associated comorbidities.

This is followed by coordinated referral to a pediatric allergist, an ENT specialist, and an orthodontist, ensuring that potentially contributing factors are evaluated within a shared multidisciplinary framework. Each specialist contributes domain-specific assessment and management while maintaining interdisciplinary communication to avoid fragmentation of care. The allergological assessment addresses allergic sensitization and nasal inflammation; the ENT evaluation focuses on nasal and adenotonsillar obstruction and on the identification of associated SDB, including objective sleep assessment when clinically indicated; and the dental/orthodontic assessment evaluates craniofacial growth, dental occlusion, and palatal morphology. Management is consequently directed toward the underlying determinants of MB, with medical, surgical, orthodontic, or functional interventions selected according to the individual clinical phenotype. Overall, this multidisciplinary pathway is intended to complement, rather than replace, established diagnostic and therapeutic recommendations for pediatric SDB and OSAS. Its added value is not the introduction of new diagnostic or therapeutic criteria for SDB/OSAS, but the use of persistent, particularly nocturnal, MB as a clinical entry point for a structured multidisciplinary assessment. While existing guidance primarily addresses children with established or suspected SDB/OSAS, the proposed framework starts from the clinical recognition of MB and links it to the systematic assessment of potentially overlapping allergic and nasal, upper airway, craniofacial, dental, and orofacial factors. By integrating these complementary perspectives from the outset, the pathway is intended to reduce the risk of overlooking coexisting contributors and to promote coordinated management and follow-up. Moreover, this approach is intended to facilitate earlier recognition of children who might otherwise remain outside conventional SDB diagnostic pathways and to promote timely referral and coordinated management across specialties. Longitudinal follow-up should be individualized according to the underlying clinical phenotype and the interventions undertaken. Reassessment should include the persistence or resolution of daytime and nocturnal MB, snoring and other sleep-related symptoms, nasal patency and allergic symptoms, treatment adherence and response, and relevant daytime manifestations. In children undergoing orthodontic management, craniofacial growth, dental occlusion, palatal morphology, and appliance adherence should also be monitored. Further objective investigations, including polysomnography, should be repeated when clinically indicated by persistent, recurrent, or newly emerging symptoms rather than routinely for all children with MB. Despite the growing evidence supporting the clinical relevance of pediatric MB, several limitations of the available literature should be acknowledged. Evidence specifically addressing persistent or nocturnal MB in children without established OSAS remains limited, and much of the available evidence derives from populations with SDB or OSAS. Therefore, the extent to which findings from these populations can be directly extrapolated to children with isolated or mild nocturnal MB remains uncertain. In addition, considerable heterogeneity exists in the definitions of MB and SDB, diagnostic criteria, assessment tools, and clinical outcomes reported across studies, limiting a direct comparison of findings. Evidence regarding the long-term effects of persistent nocturnal MB and the potential benefits of early multidisciplinary intervention is also insufficient, particularly for neurocognitive, behavioral, sleep-related, and craniofacial outcomes. Future research should therefore focus on establishing standardized definitions and diagnostic criteria for persistent and nocturnal MB, particularly in children without OSAS, and on conducting prospective longitudinal and multicenter studies to clarify its natural history and clinical consequences. Studies evaluating the effectiveness of integrated allergological, otorhinolaryngological, dental/orthodontic, and orofacial interventions are also needed. The present review has some methodological limitations. The literature search was conducted through PubMed/MEDLINE and may therefore have missed studies indexed exclusively in other databases. Reference selection followed predefined criteria of relevance, methodological robustness, and clinical applicability, but remained narrative rather than systematic. In addition, the proposed multidisciplinary pathway was developed through an evidence-informed synthesis of the available literature and was not subjected to a formal consensus process. Further prospective studies and multidisciplinary consensus will be useful to validate and refine the proposed approach.

## 7. Conclusions

MB, particularly when occurring during sleep, should not be considered merely a benign habit, but may represent an important clinical entry point for identifying nasal, upper airway, allergic, craniofacial, dental, and orofacial abnormalities, and in selected children, underlying sleep-disordered breathing (SDB). A structured multidisciplinary assessment is intended to facilitate the early recognition of these potentially overlapping conditions, particularly when nocturnal MB is accompanied by snoring, restless sleep, or other nocturnal or daytime symptoms. The proposed pathway begins with the pediatrician, who plays a central role in the initial identification of persistent or nocturnal MB and associated clinical features. Based on the initial assessment, coordinated evaluation by a pediatric allergist, ENT specialist, and orthodontist enables an assessment of the different potential determinants of MB within their respective areas of expertise. Objective assessment for SDB, including polysomnography, should be pursued when clinically indicated rather than solely on the basis of MB. The individual diagnostic and therapeutic interventions included in the pathway are based on existing evidence and current recommendations. The added value of this framework lies in its focus on persistent, particularly nocturnal, MB as the initial clinical presentation. Importantly, the pathway is intended to complement, rather than replace, established diagnostic and therapeutic recommendations for pediatric SDB and OSAS. Linking allergological, otorhinolaryngological, dental/orthodontic, and pediatric evaluations may facilitate the earlier recognition of children who might otherwise fall outside conventional SDB diagnostic pathways and promote timely, individualized, and coordinated management. Structured follow-up remains important for assessing the persistence or resolution of MB and sleep-related symptoms, as well as the response to targeted interventions, while supporting appropriate craniofacial and functional development. Further prospective studies are needed to determine whether early identification and treatment of MB, particularly in children without established OSAS, can improve long-term respiratory, sleep-related, neurobehavioral, and craniofacial outcomes.

## Data Availability

No new data were created or analyzed in this study.

## Abbreviations

The following abbreviations are used in this manuscript:

SPT	Skin Prick Testing
sIgE	Serum-Specific Immunoglobulin E
AR	Allergic Rhinitis
MB	Mouth Breathing
SDB	Sleep-Disordered Breathing
OSAS	Obstructive Sleep Apnea Syndrome
PSQ	Pediatric Sleep Questionnaire
PSG	Polysomnography
ENT	Ear, Nose and Throat
OMT	Orofacial Myofunctional Therapy
RME	Rapid Maxillary Expansion
AIT	Allergen Immunotherapy
